# Opioid overdose and cardiovascular disease-related mortality: a retrospective analysis using real-world data from the USA, 1999–2023

**DOI:** 10.3389/fcvm.2026.1771062

**Published:** 2026-05-13

**Authors:** Mohamed Fawzi Hemida, Alyaa Ahmed Ibrahim, Maryam Saghir, Ahmed Elshahat, Mohammad Rayyan Faisal, Moaz Elsayed Abouelmagd, Zeyad Kholeif, Krish Patel, Noha Hammad, Pakeezah Tabasum, Eshal Saghir, Mohammed Hammad Jaber Amin, Ahmed Elmorsy Mohamed, Khaled Ali, Basel Abdelazeem, Mustafa Al-jarshawi

**Affiliations:** 1 Faculty of Medicine, Alexandria University, Alexandria, Egypt; 2 Department of Medicine, Jinnah Sindh Medical University, Karachi, Pakistan; 3 Faculty of Medicine, Al-Azhar University, Cairo, Egypt; 4 Department of Medicine, Dow University of Health Sciences, Karachi, Pakistan; 5 Kasr-Alainy Faculty of Medicine, Cairo University, Cairo, Egypt; 6 Department of Internal Medicine, Baptist Hospital of SouthEast Texas, Beaumont, TX, United States; 7 Department of Medicine, C. U. Shah Medical College, Surendranagar, India; 8 Faculty of Medicine, Port-Said University, Port-Said, Egypt; 9 Department of Medicine, Peoples University of Medical and Health Sciences for Women, Nawabshah, Pakistan; 10 Faculty of Medicine, Alzaiem Alazhari University, Khartoum, Sudan; 11 Faculty of Medicine, Tanta University, Tanta, Egypt; 12 Department of Internal Medicine, Rosalind Franklin University of Medicine and Science, North Chicago, IL, United States; 13 Department of Cardiology, West Virginia University, Morgantown, WV, United States; 14 NIHR Academy, National Institute for Health & Care Research, Leeds, United Kingdom; 15 Keele Cardiovascular Research Group, Faculty of Medicine & Health Sciences, Keele University, Stoke-on-Trent, United Kingdom; 16 Faculty of Biology, Medicine and Health, School of Health Sciences, Division of Informatics, Imaging & Data, University of Manchester, Manchester, United Kingdom; 17 Cardiology Department, Royal Stoke University Hospitals, University Hospitals of North Midlands NHS Trust, Stoke-on-Trent, United Kingdom; 18 Institute of Health Sciences Education, Queen Mary University of London, London, United Kingdom

**Keywords:** cardiovascular disease, mortality, opioid overdose, trends, United States, disparities, epidemiology, CDC WONDER

## Abstract

**Background:**

Cardiovascular disease (CVD) is a leading cause of mortality in the U.S., a burden amplified by the ongoing opioid epidemic. This study characterized long-term trends and disparities in mortality involving both opioid overdose and CVD from 1999 to 2023.

**Methods:**

We conducted a retrospective analysis using death certificate data from the CDC WONDER multiple cause-of-death files. Data for adults aged 15 and older were analyzed using ICD-10 codes for CVD (I00-I99) and opioid overdose (T40.0, T40.1, T40.2, T40.3, T40.4). Age-adjusted mortality rates (AAMRs) per 100,000 population and crude mortality rates (CMRs) were calculated. Joinpoint regression determined the average annual percent change (AAPC).

**Results:**

A total of 112,430 deaths involving both opioid overdose and CVD were identified. The AAMR dramatically increased from 0.36 in 1999 to 4.08 in 2023 (AAPC: 10.96; 95% CI: 8.60–13.38; *p* < 0.001). Men had consistently higher mortality than women (overall AAMRs: 2.51 vs. 0.99). Non-Hispanic (NH) Black individuals showed the steepest escalation in AAMR (from 0.33 in 1999 to 6.48 in 2023; AAPC: 12.45%). The West exhibited the highest overall AAMR (2.11). Non-metropolitan areas consistently showed higher AAMRs than metropolitan areas (overall AAMR: 1.56 vs. 1.47). Middle-aged adults aged 45–64 years had the highest overall CMR (2.94).

**Conclusion:**

Opioid overdose and CVD-related mortality surged, disproportionately impacting men, NH Black individuals, Western regions, and non-metropolitan areas.

## Introduction

Cardiovascular disease (CVD) remains the leading cause of death in the United States, accounting for over a million deaths annually and projected to cause over 35 million deaths around the globe by 2050 (1, 2). Age-standardized CVD mortality fell steadily from the late 20th century through the early 2010s, driven by advances in prevention, acute care, and secondary prevention (3). However, this progress stalled and partially reversed in mid-decade, with additional surges during COVID-19 that exposed gaps in chronic disease management and access to care (4). Over the same timeframe, the USA experienced a rise in drug overdose deaths of opioids, contributing to the overall decline in life expectancy and widening sociodemographic disparities (5, 6).

The opioid epidemic has unfolded in overlapping “waves”: an initial rise related to prescription opioids, followed by heroin, and then a rapid escalation driven by illicitly manufactured fentanyl and its analogues, increasingly in combination with stimulants and other substances (5). These shifts varied by demographic and geographic factors and intersected with social determinants such as poverty, unemployment, housing instability, and limited treatment access (5–7). Similar trends have also been noticed in CVD mortality rates (3, 4). Additionally, CVD frequently contributes to mortality in opioid overdose cases (8). Studies also suggest a positive association between non-acute opioid use and overall CVD risk and mortality, especially infective endocarditis and myocardial infarction (9–11). Together, these findings highlight the complex interplay between opioid use and cardiometabolic risk.

Despite extensive work examining CVD and opioid-involved overdose mortality separately, fewer studies have characterized their long-term trajectories side-by-side using a consistent national framework through the most recent years. The Centers for Disease Control and Prevention (CDC) Wide-ranging Online Data for Epidemiologic Research (WONDER) multiple-cause-of-death files allow standardized, annually updated mortality estimation by underlying and contributing causes. This study provides a comprehensive description of long-term trends in CVD and opioid-involved overdose mortality in the United States from 1999 to 2023. The study aims to inform strategies spanning cardiovascular prevention, substance use treatment, and health-system resilience by situating two leading drivers of mortality within a single analytic frame and the most up-to-date period.

## Methods

### Study design and population

We conducted a retrospective analysis using death certificate data retrieved from the CDC WONDER database and analyzed data for adults aged 15 and older between 1999 and 2023. The study specifically examined deaths in which both opioid overdose and CVD were listed on the death certificate as either underlying or contributing causes. Opioid overdose was identified using ICD-10 codes T40.0 (opium), T40.1 (heroin), T40.2 (other opioids, including natural and semisynthetic opioids), T40.3 (methadone), and T40.4 (synthetic opioids excluding methadone), each recorded as multiple causes of death. CVD was identified using ICD-10 codes I00–I99 (diseases of the circulatory system), also recorded as multiple causes of death. These ICD codes have been previously used to identify cardiovascular conditions and opioid overdoses in administrative datasets (12, 13). This age threshold was carefully chosen after a thorough assessment of data availability and reliability within the CDC WONDER database. Specifically, preliminary explorations revealed that mortality data for individuals under 15 years often exhibited significant suppression due to small cell counts across various demographic and geographic variables. Including these suppressed data points would have led to unreliable estimates and incomplete trend analyses, particularly for assessing disparities. By focusing on the ≥15 age group, we ensured the statistical robustness and completeness of the dataset for our comprehensive analysis of opioid overdose and CVD-related mortality trends and disparities. Institutional review board approval was not required because the study used de-identified, publicly accessible data and adhered to the Strengthening the Reporting of Observational Studies in Epidemiology (STROBE) guidelines (14).

### Data abstraction

Data for population size and demographics, such as sex, age, race, and region were extracted. The place of death was categorized into medical facilities, hospice, home, and nursing home/long-term care facilities. Racial and ethnic categories were classified as non-Hispanic (NH) White, NH Black or African American, and Hispanic or Latino. The National Center for Health Statistics Urban-Rural Classification Scheme was used to assess the population by urban (where the large metropolitan area had a population ≥1 million, whereas medium/small metropolitan areas had a population of 50,000–999,999) and rural (population <50,000) counties per the 2013 U.S. census classification (15). It is important to note that urban-rural data were consistently available and analyzed only for the period 1999–2020 due to historical limitations in WONDER stratifications. Regions were stratified into Northeast, Midwest, South, and West according to the U.S. Census Bureau definitions (16). For age-stratified analyses, decedents were categorized into three predefined age groups: 15–44 years, 45–64 years, and 65 years and older.

### Statistical analysis

Crude and age-adjusted mortality rates (CMRs and AAMRs) per 100,000 population from 1999 to 2023 by year, sex, race/ethnicity, state, and urban-rural status for the years 1999–2020 with 95% CIs were calculated, using the 2000 U.S. population as the standard (17). CMRs were determined by dividing the number of CVD and opioid overdose deaths by the corresponding U.S. population for each year, with 95% confidence intervals (CIs) computed for all estimates.

To evaluate national temporal trends in CMRs and AAMRs, we used the Joinpoint Regression Program (Version 5.4.0.0; National Cancer Institute) (18). Consistent with established methodology, log-transformed mortality rates were modeled using log-linear regression, allowing the identification of changes in trend where statistically significant inflection points occurred.

A maximum of four joinpoints was tested to balance model flexibility with parsimony, in accordance with NCI recommendations for datasets of similar length. Model selection was performed using the Monte Carlo permutation method, and Bonferroni corrections were applied to adjust for multiple comparisons across tested models.

For each identified segment, we calculated the Annual Percent Change (APC) with corresponding 95% CIs. APCs were classified as increasing or decreasing when the slope was significantly different from zero using a two-sided *t*-test. Joinpoint regression identifies time points (“joinpoints”) where statistically significant changes in mortality trends occur. Each segment reflects a distinct period with its own APC, allowing interpretation of accelerations or decelerations in trends. To summarize overall mortality trends across the full study period, the Average Annual Percent Change (AAPC) was computed, also presented with 95% CIs. A *p*-value <0.05 was considered statistically significant.

## Results

### Overall trends

**Figure 1:**
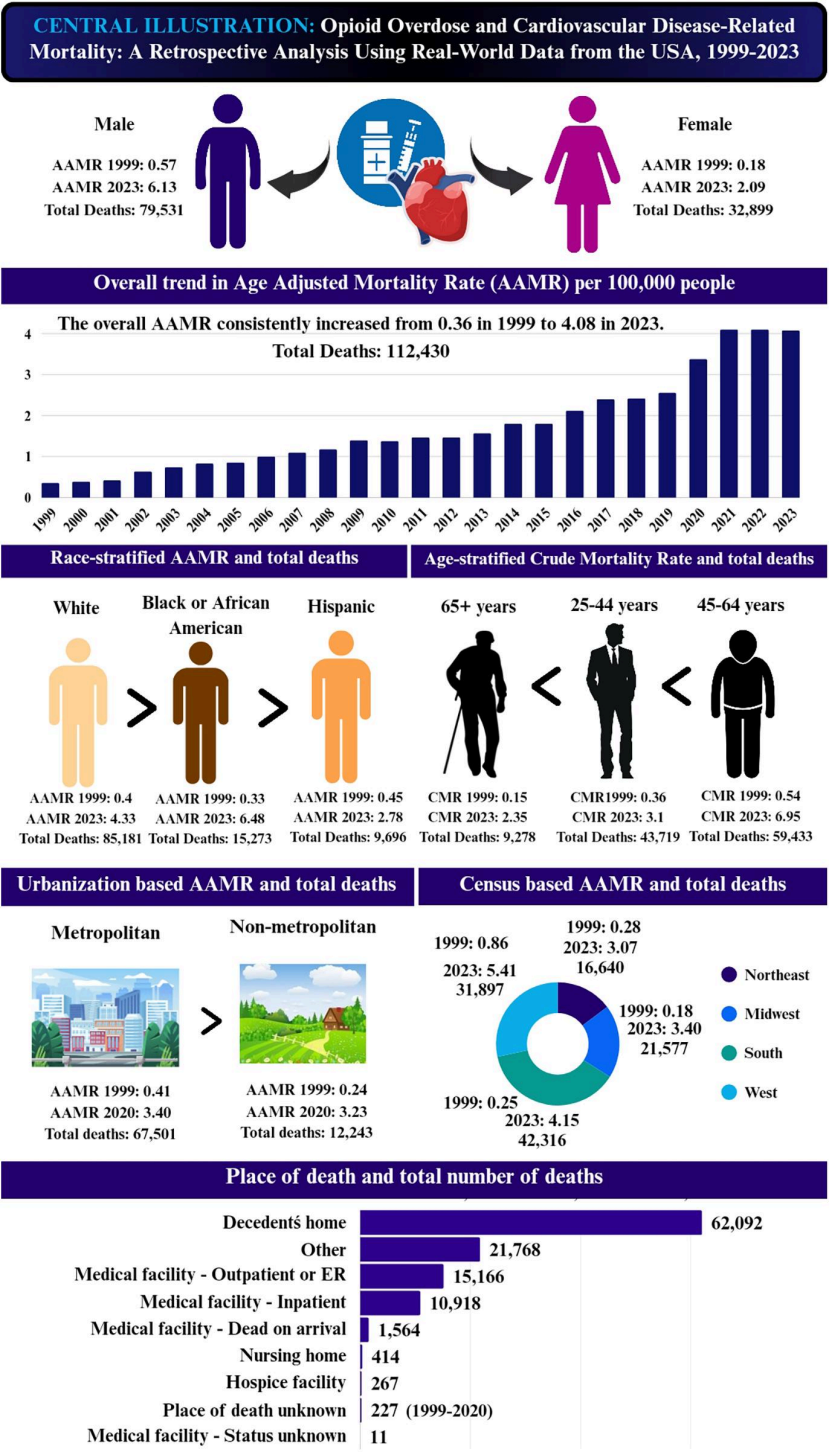
Central Illustration has been created by us using Canva.

Throughout the study timeframe, from 1999 to 2023, a total of 112,430 deaths resulted from opioid overdose and CVD. The AAMR observed an overall marked rise during the study period, from 0.36 (95% CI: 0.34–0.39) in 1999 to 4.08 (95% CI: 4.01–4.16) in 2023, with an AAPC of 10.96 (95% CI: 8.60 to 13.38; *p* < 0.001) (Supplementary Tables S1, S2; Figure 1).

**Figure 2:**
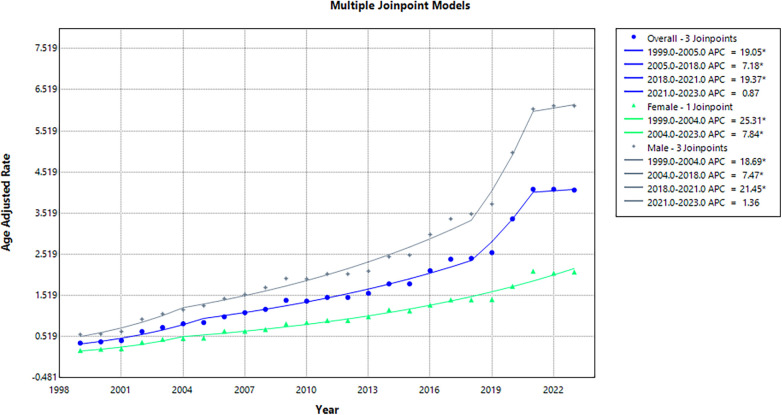
Annual Age-adjusted mortality rates (AAMR) by overall and Sex, 1999–2023.

From 1999 to 2005, AAMR demonstrated a subtle yet significant rise, from 0.36 to 0.86 (APC: 19.05; 95% CI: 13.27 to 25.14; *p* = 0.003). This was followed by a subsequent significant incline to 2.42 in 2018 (APC: 7.18; 95% CI: 6.06 to 8.32; *p* < 0.001), a significant surge to 4.1 in 2021 (APC: 19.37; 95% CI: 4.41 to 36.48; *p* = 0.013), and a non-significant drop to 4.08 in 2023 (APC: 0.87; 95% CI: −9.75 to 12.73; *p* = 0.86) (Supplementary Tables S1–S3; Figure 2).

### Sex trends

Between 1999 and 2023, men exhibited higher mortality than women (79,531 vs. 32,899 deaths). Accordingly, the AAMR remained consistently higher in men, with overall AAMRs of 2.51 and 0.99 for men and women, respectively. In 1999, the AAMR for men was 0.57 (95% CI: 0.52–0.61) compared to 0.18 (95% CI: 0.15–0.2) in women. These values increased to 6.13 (95% CI: 5.99–6.26) and 2.09 (95% CI: 2.01–2.17) by the year 2023. However, women exhibited a greater AAPC of 11.27 (95% CI: 9.25 to 13.33; *p* < 0.001), compared to men with an AAPC of 10.67 (95% CI: 8.32 to 13.46; *p* < 0.001).

AAMR among women rose significantly, from 0.18 in 1999 to 0.47 in 2004 (APC: 25.31; 95% CI: 14.46 to 37.19; *p* = 0.004), followed by a continued marked rise to 2.09 in 2023 (APC: 7.84; 95% CI: 7.16 to 8.54; *p* < 0.001). In men, AAMR saw a significant increase from 0.57 in 1999 to 1.17 in 2004 (APC: 18.69; 95% CI: 10.28 to 27.75; *p* = 0.001), followed by a steady yet significant rise to 3.5 in 2018 (APC: 7.47; 95% CI: 6.30 to 8.65; *p* < 0.001), a significant surge to 6.05 in 2021 (APC: 21.44; 95% CI: 6.46 to 38.55; *p* = 0.006), and a slight but non-significant increase to 6.13 in 2023 (APC: 1.36; 95% CI: −9.95 to 14.09; *p* = 0.81) (Supplementary Tables S1–S3; Figure 2).

### Racial trends

When stratified by race, NH White individuals accounted for the highest number of deaths (85,181), followed by NH Black/African American individuals (15,273), and Hispanic/Latino (9,696). Consequently, NH White individuals demonstrated the greatest overall AAMR of 2.08, followed by NH Black/African American individuals (1.82) and Hispanic/Latino individuals (0.98).

All races observed a significant increase in AAMR between 1999 and 2023; 0.40 to 4.33 (AAPC: 11.21; 95% CI: 8.68 to 13.80; *p* < 0.001) in NH White individuals, 0.33 to 6.48 (AAPC: 12.45; 95% CI: 10.12 to 14.83; *p* < 0.001) among NH Black/African American individuals, and 0.45 to 2.78 (AAPC: 9.16; 95% CI: 7.96 to 13.37; *p* < 0.001) among Hispanic/Latino individuals.

**Figure 3:**
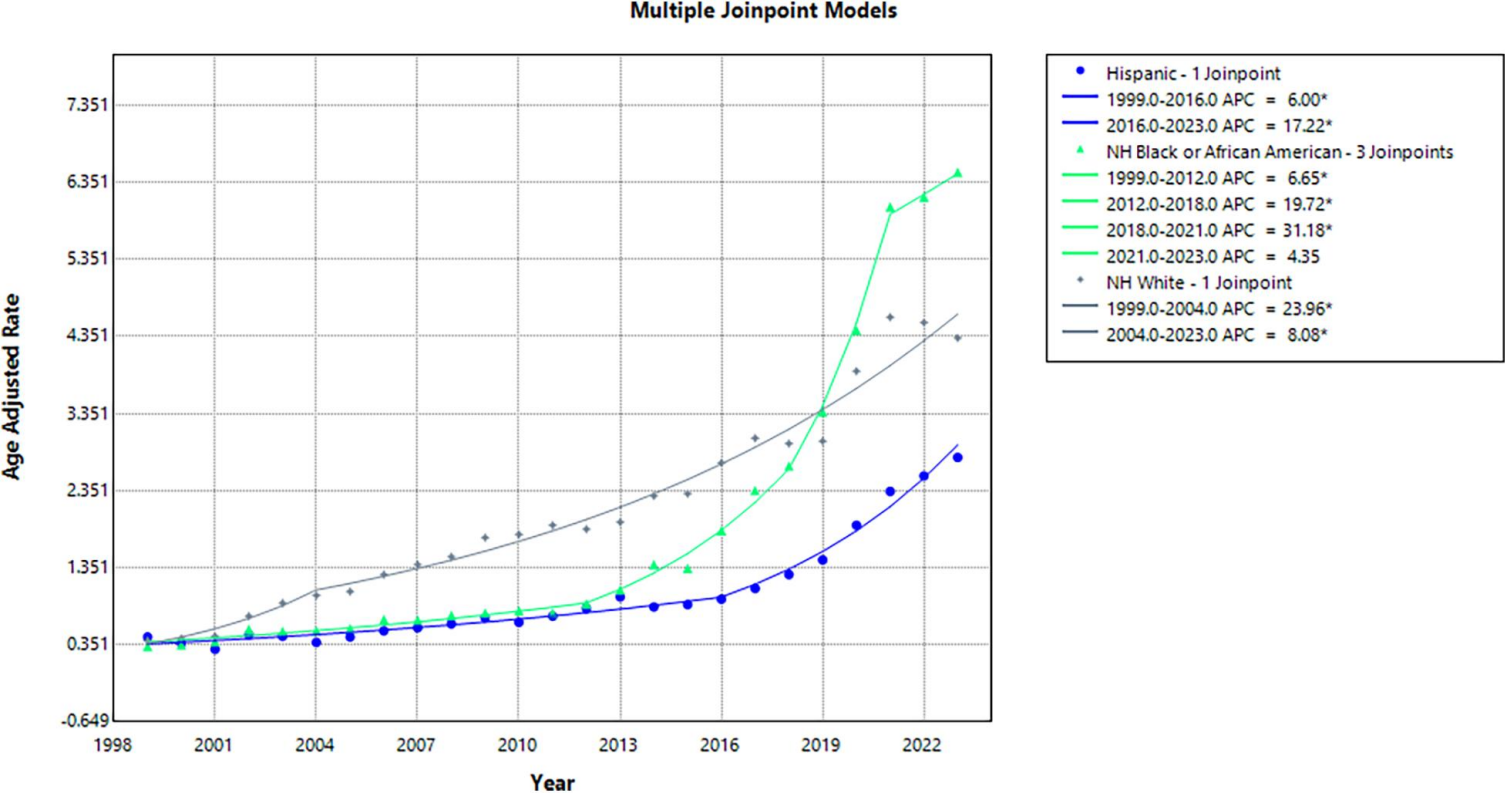
Annual Age-adjusted mortality rates (AAMR) by race/ethnicity, 1999–2023.

In brief, NH Black/African American individuals experienced a moderate yet significant increase in AAMR from 0.33 in 1999 to 0.88 in 2012 (APC: 6.65; 95% CI: 4.62 to 8.72; *p* = 0.005), followed by a marked increase to 2.67 in 2018 (APC: 19.72; 95% CI: 14.52 to 25.16; *p* = 0.001), a continued upward trend to 6.03 in 2021 (APC: 31.18; 95% CI: 16.16 to 48.14; *p* = 0.002), and a non-significant increase to 6.48 in 2023 (APC: 4.35; 95% CI: −5.10 to 14.73; *p* = 0.35). NH White individuals demonstrated a significant rise in AAMR throughout the study period, initially from 0.40 in 1999 to 0.99 in 2004 (APC: 23.96; 95% CI: 10.56 to 39.00; *p* = 0.008), followed by a continued increase, reaching 4.33 in 2023 (APC: 8.08; 95% CI: 7.31 to 8.85; *p* < 0.001). Similarly, Hispanic/Latino individuals exhibited an initial significant incline in AAMR from 0.45 in 1999 to 0.94 in 2016 (APC: 6.00; 95% CI: 4.56 to 7.46; *p* < 0.001), followed by a significant surge to 2.78 in 2023 (APC: 17.22; 95% CI: 14.57 to 19.94; *p* < 0.001) (Supplementary Tables S1, S2, S4; Figure 3).

### Regional trends

Between 1999 and 2023, the Southern region reported the highest number of deaths (42,316), followed by the West (31,897), the Midwest (21,577), and the Northeast (16,640). Overall, AAMRs were highest in the West (2.11), followed by the South (1.76), the Midwest (1.62), and the Northeast (1.44).

All regions demonstrated an overall increase in AAMR between 1999 and 2023. In the Northeast, AAMR increased from 0.28 to 3.07 (AAPC: 10.55; 95% CI: 8.82 to 12.30; *p* < 0.001), while the Midwest saw a rise from 0.18 to 3.40 (AAPC: 12.81; 95% CI: 8.55 to 17.24; *p* < 0.001). The West experienced an incline from 0.86 to 5.41 (AAPC: 9.01; 95% CI: 6.99 to 11.06; *p* < 0.001), and the South saw an increase from 0.25 to 4.15 (AAPC: 11.77; 95% CI: 9.39 to 14.20; *p* < 0.001) over the same period.

AAMR in the Northeast exhibited an initial, significant incline, from 0.28 in 1999 to 3.32 in 2021 (APC: 11.92; 95% CI: 10.97 to 12.87; *p* < 0.001), followed by a non-significant drop to 3.07 in 2023 (APC: −3.42; 95% CI: −19.19 to 15.42; *p* = 0.68). In the Midwest, AAMR rose significantly from 0.18 in 1999 to 0.54 in 2002 (APC: 42.63; 95% CI: 4.34 to 94.97; *p* = 0.02), followed by a significant increase to 3.72 in 2021 (APC: 10.36; 95% CI: 9.49 to 11.23; *p* < 0.001), and a slight yet non-significant drop to 3.40 in 2023 (APC: −2.20; 95% CI: −15.77 to 13.56; *p* = 0.75).

**Figure 4:**
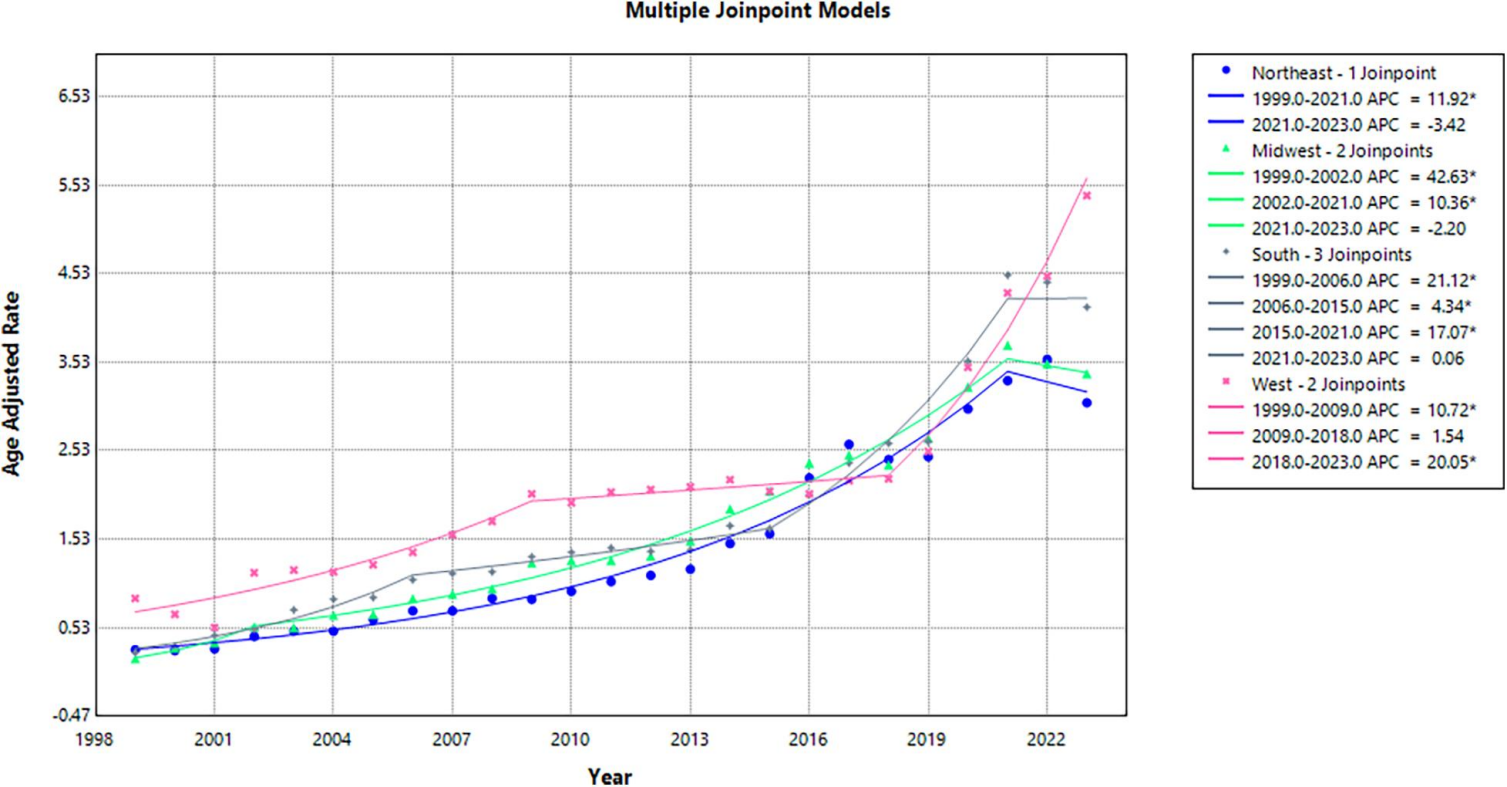
Regional trends in Age-adjusted mortality rates (AAMR), 1999–2023.

In the Southern region, AAMR increased significantly from 0.25 in 1999 to 1.07 in 2006 (APC: 21.12; 95% CI: 14.75 to 27.84; *p* = 0.002), followed by a significant upward trend, reaching 1.65 in 2015 (APC: 4.34; 95% CI: 1.63 to 7.13; *p* = 0.003), a significant surge to 4.51 in 2021 (APC: 17.07; 95% CI: 12.79 to 21.50; *p* < 0.001), and a steady but non-significant decrease to 4.15 in 2023 (APC: 0.06; 95% CI: −12.47 to 14.38; *p* = 0.99). The Western region experienced a marked initial incline in AAMR from 0.86 in 1999 to 2.04 in 2009 (APC: 10.72; 95% CI: 7.20 to 14.30; *p* = 0.004), followed by a continued rise to 2.21 in 2018 (APC: 1.54; 95% CI: −1.58 to 4.77; *p* = 0.31), and a significant surge to 5.41 in 2023 (APC: 20.05; 95% CI: 14.81 to 25.52; *p* < 0.001) (Supplementary Table S1, S2, S5; Figure 4).

### Place of death

Between 1999 and 2023, place of death data were available for 112,430 cases. The majority of deaths occurred in the decedent's home (55.23%), followed by medical facilities (24.60%), nursing homes or long-term care facilities (0.37%), and hospice settings (0.24%). A small proportion of deaths (0.20%) occurred in unspecified locations, while the remaining proportion of deaths (19.36%) occurred in other locations (Supplementary Table S6).

### Urbanization trends

Between 1999 and 2020, metropolitan areas accounted for a higher number of deaths (67,501) compared to non-metropolitan areas (12,243). From 1999 to 2020, both metropolitan and non-metropolitan areas demonstrated a rise in AAMR. AAMR in metropolitan areas rose from 0.41 to 3.4 (AAPC: 11.17; 95% CI: 5.57 to 14.90; *p* < 0.001), compared to non-metropolitan areas, which increased from 0.24 to 3.23 (AAPC: 12.01; 95% CI: 9.25 to 14.83; *p* < 0.001). The overall AAMR remained higher in non-metropolitan areas (1.56) relative to metropolitan areas (1.47).

**Figure 5:**
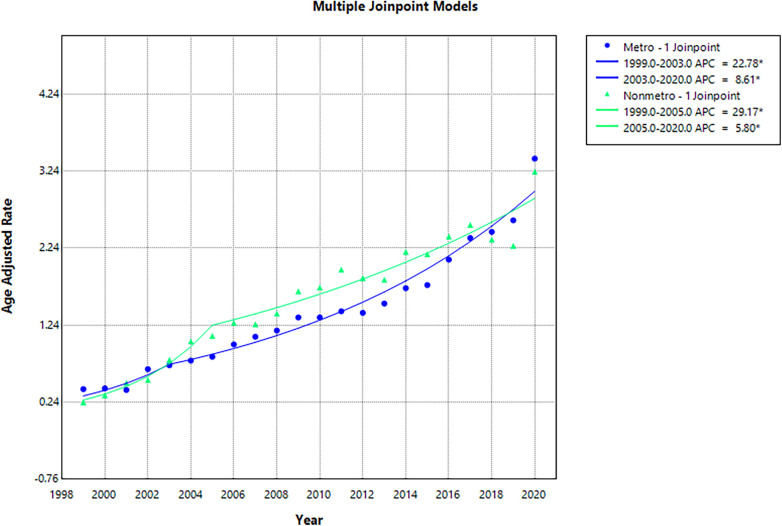
Age-Adjusted mortality rates (AAMR) by urbanization, 1999–2020.

Metropolitan regions experienced a significant increase in AAMR from 0.41 in 1999 to 0.72 in 2003 (APC: 22.78; 95% CI: 2.27 to 47.39; *p* = 0.029), followed by a continued significant rise to 3.40 in 2020 (APC: 8.61; 95% CI: 7.68 to 9.55; *p* < 0.001). In non-metropolitan areas, AAMR rose significantly from 0.24 in 1999 to 1.1 in 2005 (APC: 29.17; 95% CI: 18.05 to 41.34; *p* = 0.001), followed by a significant rise to 3.23 in 2020 (APC: 5.80; 95% CI: 4.68 to 6.93; *p* < 0.001) (Supplementary Tables S2, S7; Figure 5).

### Age-Specific trends

From 1999 to 2023, middle-aged adults aged 45–64 years accounted for the highest number of deaths (59,433), followed by young-aged adults 15–44 years (43,719) andolder adults aged ≥65 years (9,278). Across the study period (1999–2023), all age groups experienced an incline in CMR. Among older adults, CMR increased from 0.15 in 1999 to 2.35 in 2023 (AAPC: 12.31; 95% CI: 11.32 to 13.31; *p* < 0.001). Young adults also showed a significant rise in CMR, from 0.36 to 3.1 over the same period (AAPC: 8.47; 95% CI: 5.33 to 11.70; *p* = 0.02). CMR among middle-aged adults rose from 0.54 to 6.95 (AAPC: 10.83; 95% CI: 9.01 to 12.68; *p* < 0.001).

Middle-aged adults aged 45–64 years exhibited the greatest overall CMR (2.94), followed by young-aged adults 15–44 years (1.37), and older adults aged ≥65 years (0.74). Among young adults, the CMR rose significantly from 0.36 in 1999 to 1.86 in 2018 (APC: 7.81; 95% CI: 6.68 to 8.96; *p* < 0.001), followed by a non-significant increase to 3.32 in 2021 (APC: 21.21; 95% CI: −2.04 to 48.98; *p* = 0.073), and a subsequent non-significant decline to 3.10 in 2023 (APC: −2.71; 95% CI: −18.49 to 16.13; *p* = 0.74). Among middle-aged adults, the CMR rose significantly from 0.54 in 1999 to 1.89 in 2007 (APC: 16.13; 95% CI: 12.88 to 19.47; *p* < 0.001), followed by a significant surge to 4.14 in 2018 (APC: 6.68; 95% CI: 5.56 to 7.82; *p* < 0.001), a continued upward trend to 6.77 in 2021 (APC: 18.26; 95% CI: 6.75 to 31.0; *p* = 0.003), and a non-significant increase to 6.95 in 2023 (APC: 2.94; 95% CI: −5.55 to 12.19; *p* = 0.48).

**Figure 6:**
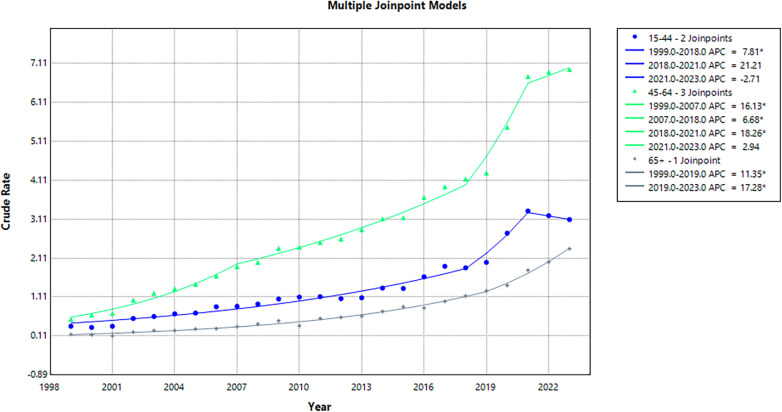
Age-Specific crude mortality rates (CMR) for opioid overdose and CVD, 1999–2023.

In older adults, a significant incline in CMR was observed from 0.15 in 1999 to 1.27 in 2019 (APC: 11.35; 95% CI: 10.40 to 12.30; *p* < 0.001), followed by a significant rise to 2.35 in 2023 (APC: 17.28; 95% CI: 13.00 to 21.73; *p* < 0.001) (Supplementary Tables S2, S8; Figure 6).

## Discussion

This retrospective study identifies 112,430 deaths between 1999 and 2023 in which both opioid overdose and CVD were recorded. The AAMR increased nearly twelvefold, from 1999 to 2023, with distinct inflection points reflecting the broader opioid epidemic. There were initial modest increases that gave way to a steady rise through 2018 (AAMR 2.42), followed by a marked escalation to 4.10 in 2021 before stabilizing. Men accounted for the majority of deaths and persistently revealed higher AAMRs overall. When stratified by race, NH White individuals showed the most significant absolute burden. In the same context, the NH Black individuals exhibited around a twentyfold increase in death rates. Geographically, the South recorded the highest absolute number of deaths, whereas the West exhibited the highest mortality rate. Non-metropolitan areas showed persistently higher AAMRs than metropolitan areas. Over half of deaths occurred at home, underscoring the sudden, unattended nature of events. Age-specific analyses revealed the greatest death rates among middle-aged adults, with substantial increases also seen in younger and older adults.

Opioid overdose represents a leading cause of premature mortality in the United States, and its lethality is commonly associated with cardiovascular collapse. Mechanistically, opioid overdose causes profound respiratory depression, resulting in severe hypercapnia and hypoxemia, and rapidly precipitating cardiac arrest (19, 20). Notably, the incidence of overdose-related cardiac arrest rose by more than double from 2015 to 2021 (5.2 vs. 13 per 100,000 person-years), with mortality exceeding 80% despite advanced resuscitation protocols (21). Certain opioids confer elevated mortality rates through cardiac-specific toxicities. For instance, synthetic opioids like fentanyl are strongly implicated in escalating mortality trends as they have robust potency, rapid onset, and high risk of apnea-related death (22, 23). Methadone showed a dose-dependent QT prolongation and torsades de pointes that may lead to sudden cardiac death, with population-based estimates reporting around 14 methadone-induced sudden deaths per 100,000 person-years (19, 24, 25). Beyond overdose-related fatalities, chronic opioid consumption may further amplify cardiovascular mortality rates via mechanisms encompassing arrhythmia, myocardial ischemia, and infective endocarditis. Supporting this, prior research indicates an elevated risk of fatal cardiovascular events among opioid users, even after controlling for comorbidities (20, 26).

Our CDC-WONDER analysis extends these observations, demonstrating that opioid and CVD-related mortality have escalated markedly over the past 25 years. The AAMR rates increased around twelvefold from 1999 to 2023, with APC exceeding 19% in specific inflection periods. We observed distinct temporal inflection points in mortality, which were consistent with prior U.S. epidemiological reports (27). Early escalations in the 2,000 s were modest but accelerated considerably after 2010, paralleling the broader opioid epidemic characterized by prescription opioid misuse, subsequent heroin use, and the later predominance of synthetic opioids, primarily fentanyl (28). In the same context, the steepest escalation occurred after 2020, coinciding with the widespread infiltration of illegally manufactured fentanyls and potent analogs like carfentanil into the drug supply (29). These medications, due to their high potency and unpredictable dosing, have been significantly associated with fatal overdoses. Additionally, this period coincided with the COVID-19 pandemic, which hindered healthcare access and exacerbated vulnerability in populations with preexisting cardiovascular disease, further increasing mortality risk (12, 30).

Although synthetic opioids accounted for the highest absolute increase after 2020, recent years exhibited a plateau in AAMRs. This stabilization may reflect increased availability of naloxone, harm reduction strategies, and state-level policy measures developed to mitigate fentanyl-related fatalities (31). Nevertheless, despite this slight equalization, the overall burden remains elevated in patients with cardiovascular disease, indicating that cardiovascular impairment may be associated with increased mortality risk of opioid overdoses through mechanisms such as arrhythmogenesis, myocardial depression, and compromised autonomic regulation. Collectively, our findings highlight that the increase and partial stabilization of opioid-related mortality in cardiovascular disease populations are not merely epidemiological reflections of national trends but also clinically amplified by the pathophysiological vulnerabilities associated with CVD.

In both national trend analyses and state-level investigations, men consistently demonstrated higher overdose mortality than women (27, 32). Supporting this, our analysis highlighted that males accounted for the majority of deaths and exhibited higher overall AAMRs than females. In their state-level analysis, Butelman et al. (32) emphasized that this disparity is not incidental but consistent across drug categories and regions. They found that male-to-female overdose mortality ratios were strikingly stable: 2.5 for synthetic opioids, 2.9 for heroin, 2.4 for psychostimulants, and 2.8 for cocaine. At the national level, synthetic opioid mortality reached 37.4 per 100,000 among men compared with 13.9 per 100,000 among women, with a ratio of 2.7 (32). Notably, these estimates remained consistent even after adjusting for sex-specific drug-misuse rates, highlighting that the elevated prevalence of opioid use alone among males cannot explain this disparity. Possible explanations for this distinction include that men have a greater propensity for risk-taking than females, a pattern mirrored in other injury-related deaths, such as motor vehicle accidents (33).

Our analysis demonstrated considerable racial disparities, with NH White individuals bearing the highest absolute number of deaths and the greatest overall AAMR. Nevertheless, when considering temporal inflections, NH Black/African American individuals experienced the highest escalation, especially after 2012. Their AAMR rose markedly from 2012 to 2021, before stabilization in 2023. This sharp elevation contrasts with the more gradual, though consistently significant, increase noticed among NH White and Hispanic/Latino individuals. Consistent findings were reported by Smith et al. (34), who observed notable racial disparities in recent years, with NH Black communities disproportionately impacted by the fentanyl's spread compared with other races. Smith et al. (34) observed that in specific regional “epicenters”, encompassing the upper Midwest, NH Black and Native populations encountered mortality rates comparable or exceeding those of NH White populations, despite historically lower burden. These distinctions were attributed not only to fentanyl's spread into urban drug markets but also to systemic inequities, such as limited access to harm-reduction resources and lower retention in Office-Based Addiction Treatment programs compared to White people (35, 36).

Moreover, clear regional disparities were observed in our national trend analysis. The West revealed the greatest AAMR by 2023, escalating steadily across the study period. This trajectory aligns with fentanyl market dynamics unique to this region, in which illegally manufactured fentanyls (IMFs) entered later but spread rapidly through counterfeit pills, resulting in a 33.9% increase in IMF-related deaths between 2022 and 2023 (29). In contrast, the Northeast and Midwest exhibited plateaus or declines after 2021, aligning with regional IMF mortality decreases (Northeast: −3.2%, Midwest: −7.8%) (29). The South, which recorded the highest number of deaths (42,316) but lower overall AAMRs compared with the West, reflects earlier IMF penetration outside western states and more recent national deceleration in opioid mortality (29).

Our findings highlight critical contextual patterns when stratified by place of death and urbanization. More than half of the fatalities occurred within the home, reflecting the persistent challenge of timely detection and intervention in private settings. Importantly, the relatively small proportion of deaths within medical facilities (24.6%) could be justified by the fact that many patients do not survive long enough to receive medical care, which emphasizes the need for earlier recognition, bystander intervention, and prehospital naloxone administration. Analysis by urbanization level indicated that while metropolitan regions encountered a greater absolute number of opioid overdose and CVD-related deaths, non-metropolitan areas exhibited persistently higher AAMR. These findings corroborate national evidence of rural-urban disparities in overdose mortality, reflecting significant barriers to harm reduction services and timely emergency care in rural settings.

Age-specific analyses demonstrated the highest mortality burden among middle-aged adults, consistent with national epidemiological trends that identify this group as a particularly vulnerable group to opioid-related harms, owing to the high prevalence of prescription opioid exposure, polysubstance use, and socioeconomic vulnerability (27, 37). Notably, mortality among older adults has increased steeply in recent years, a pattern that may reflect elevated opioid prescribing for chronic pain and heightened physiological susceptibility to respiratory depression and overdose in this population (34, 37). While younger adults also experienced significant increases in overdose mortality, their more recent plateau suggests possible effects of targeted prevention strategies, expanded naloxone availability, and evolving substance use patterns, including substitution with stimulants or non-opioid drugs.

### Strengths and limitations

This study provides one of the most comprehensive longitudinal analyses of opioid and CVD-related mortality in the United States from 1999 to 2023. The large sample size, encompassing diverse demographic, geographic, and subgroups, supports the generalizability and enhances the reliability of our conclusions. A key strength is the utilization of Joinpoint regression, which enables robust identification of temporal inflection points and quantification of changes in mortality trends.

Nevertheless, this study has critical limitations that must be acknowledged. First, the study design is ecological and descriptive, hindering causal inference regarding the observed associations between cardiovascular disease and opioid overdose mortality. Death certificate data do not allow the determination of the temporal sequence between opioid overdose and CVD, and it cannot be established whether CVD preceded, contributed to, or resulted from the overdose event. Second, opioid overdose was identified using multiple cause-of-death fields and included cases in which opioid overdose was listed either as the underlying cause of death or as a contributing cause. These categories were combined in the analysis. While this approach increases sensitivity for identifying opioid involvement, it may also introduce heterogeneity, as the clinical context and causal role of opioid exposure may differ between underlying and contributing causes. Also, we excluded ICD-10 code T40.6 (other and unspecified narcotics) from the analysis to enhance diagnostic specificity and minimize exposure misclassification, as this code may include non-opioid narcotics or cases in which the specific substance was not clearly identified on the death certificate.

Third, the reliability of CDC WONDER data depends on the accuracy and completeness of ICD coding. Misclassification of both opioid involvement and cardiovascular conditions is possible, particularly in cases of polysubstance use or limited toxicological testing. Fourth, death certificate data lack individual-level clinical detail. Information on comorbidities, prior cardiovascular history, medication use (including opioid prescriptions or treatment for opioid use disorder), toxicology concentrations, and acute clinical circumstances is unavailable. The absence of these data limits adjustment for confounding factors and prevents more granular risk stratification. Additionally, variability in toxicological testing and reporting practices across states may affect the detection of opioid involvement, and the dataset does not allow differentiation between acute overdose events and chronic opioid exposure. Fifth, we excluded the racial/ethnic groups “Non-Hispanic American Indian or Alaska Native” and “Non-Hispanic Asian or Pacific Islander” from the analysis due to data suppression in the CDC WONDER database, which limited reliable reporting for these populations.

### Clinical implications and future directions

These findings underscore that opioid involvement is frequently observed among individuals with coexisting CVD. This co-occurrence may reflect shared risk pathways, including acute cardiopulmonary effects and underlying cardiovascular vulnerability, rather than a direct causal relationship. Clinically, this highlights the necessity for integrated care models that link cardiovascular prevention with substance use treatment and harm reduction strategies. Screening for opioid use, provision of naloxone, and safer prescribing practices should be prioritized in patients with established CVD.

Future research should move beyond descriptive epidemiology to mechanistic and longitudinal designs that explore how chronic opioid exposure contributes to cardiovascular pathology and mortality. Linking administrative mortality data with clinical registries and electronic health records could clarify pathways of risk and improve surveillance. In addition, the potential impact of evolving state and federal policies should be considered when interpreting mortality trends. Changes in opioid prescribing regulations may have reduced access to prescription opioids but may also have contributed to shifts toward illicit or synthetic opioids, including fentanyl and its analogues, which are associated with greater potency and potentially higher cardiovascular toxicity and overdose risk. Variations in harm-reduction policies, naloxone access, and medication treatment availability across states may further influence geographic disparities. Broader structural factors, including immigration and socioeconomic policies, may also shape vulnerability, healthcare access, and risk environments in certain communities. Evaluating the cardiovascular implications of these policy environments, alongside harm-reduction and treatment strategies, will be essential. Interdisciplinary approaches that integrate cardiology, addiction medicine, and public health are urgently needed to mitigate the intersecting burdens of cardiovascular disease and opioid-related mortality.

## Conclusion

Opioid overdose and CVD-related mortality have risen markedly in the United States over the past 25 years, with distinct demographic, regional, and temporal disparities. While recent stabilization may suggest partial progress, the overall burden remains high. Integrated, equity-focused strategies combining cardiovascular care with substance use prevention and harm reduction are critical to reduce mortality and address the intersecting vulnerabilities driving this dual epidemic.

## Data Availability

This study used publicly available, de-identified data from the CDC WONDER database.
