# Supplementary material for: Opioid overdose and cardiovascular disease-related mortality: a retrospective analysis using real-world data from the USA, 1999–2023
Source: Front Cardiovasc Med. 2026 May 13;13:1771062. doi: 10.3389/fcvm.2026.1771062 (PMC13212144; doi:10.3389/fcvm.2026.1771062)
Supplement: Supplementary file 1 [file Supplementaryfile1.docx]

**Supplemental Table 1** Cardiovascular and Opioids Overdose–related Deaths, Stratified by Sex and Race, in Adults in the United States, 1999 to 2023.

| **Year** | **Overall** | **Men** | **Women** | **NH White** | **NH Black** | **Hispanic/ Latino** |
| --- | --- | --- | --- | --- | --- | --- |
| 1999 | 822 | 617 | 205 | 637 | 80 | 100 |
| 2000 | 861 | 616 | 245 | 695 | 81 | 79 |
| 2001 | 942 | 689 | 253 | 756 | 105 | 70 |
| 2002 | 1,458 | 1,013 | 445 | 1,181 | 142 | 112 |
| 2003 | 1,689 | 1,186 | 503 | 1,405 | 135 | 114 |
| 2004 | 1,882 | 1,316 | 566 | 1,609 | 145 | 94 |
| 2005 | 2,037 | 1,453 | 584 | 1,719 | 155 | 126 |
| 2006 | 2,435 | 1,640 | 795 | 2,054 | 196 | 148 |
| 2007 | 2,682 | 1,853 | 829 | 2,272 | 197 | 171 |
| 2008 | 2,907 | 2,038 | 869 | 2,458 | 214 | 192 |
| 2009 | 3,425 | 2,332 | 1,093 | 2,915 | 231 | 229 |
| 2010 | 3,494 | 2,352 | 1,142 | 2,961 | 252 | 222 |
| 2011 | 3,724 | 2,523 | 1,201 | 3,162 | 231 | 258 |
| 2012 | 3,756 | 2,545 | 1,211 | 3,103 | 281 | 291 |
| 2013 | 4,020 | 2,665 | 1,355 | 3,257 | 344 | 352 |
| 2014 | 4,664 | 3,096 | 1,568 | 3,797 | 454 | 324 |
| 2015 | 4,773 | 3,219 | 1,554 | 3,897 | 449 | 350 |
| 2016 | 5,600 | 3,869 | 1,731 | 4,488 | 619 | 385 |
| 2017 | 6,296 | 4,355 | 1,941 | 4,937 | 787 | 449 |
| 2018 | 6,486 | 4,571 | 1,915 | 4,896 | 927 | 546 |
| 2019 | 6,879 | 4,909 | 1,970 | 4,941 | 1,172 | 647 |
| 2020 | 8,912 | 6,499 | 2,413 | 6,296 | 1,547 | 866 |
| 2021 | 10,797 | 7,930 | 2,867 | 7,409 | 2,088 | 1,074 |
| 2022 | 10,876 | 8,075 | 2,801 | 7,296 | 2,140 | 1,171 |
| 2023 | 11,013 | 8,170 | 2,843 | 7,040 | 2,301 | 1,326 |
| **Total** | 112,430 | 79,531 | 32,899 | 85,181 | 15,273 | 9,696 |

NH, non-Hispanic

**Supplemental Table 2** Annual percent change (APC) of Cardiovascular and Opioids Overdose–related Age-Adjusted Mortality Rates per 100,000 in Adults in the United States, 1999 to 2023.

| **Year Interval** | **APC (95% CI)** |
| --- | --- |
| **Overall** |  |
| 1999-2005 | 19.05 (13.26 to 25.13) |
| 2005-2018 | 7.18 (6.05 to 8.32) |
| 2018-2021 | 19.37 (4.41 to 36.47) |
| 2021-2023 | 0.86 (−9.7 to 12.73) |
| **Women** |  |
| 1999-2004 | 25.31 (14.4 to 37.19) |
| 2004-2023 | 7.84 (7.15 to 8.53) |
| **Men** |  |
| 1999-2004 | 18.69 (10.28 to 27.74) |
| 2004-2018 | 7.47 (6.3 to 8.64) |
| 2018-2021 | 21.44 (6.46 to 38.54) |
| 2021-2023 | 1.35 (−9.95 to 14.08) |
| **NH White** |  |
| 1999-2004 | 23.96 (10.55 to 38.99) |
| 2004-2023 | 8.07 (7.31 to 8.84) |
| **NH Black** |  |
| 1999-2012 | 6.65 (4.62 to 8.72) |
| 2012-2018 | 19.72 (14.52 to 25.16) |
| 2018-2021 | 31.17 (16.16 to 48.13) |
| 2021-2023 | 4.34 (−5.09 to 14.73) |
| **Hispanic/ Latino** |  |
| 1999-2016 | 5.99 (4.55 to 7.45) |
| 2016-2023 | 17.22 (14.56 to 19.93) |
| **Metropolitan** |  |
| 1999-2003 | 22.77 (2.26 to 47.39) |
| 2003-2020 | 8.6 (7.67 to 9.54) |
| **Non-metropolitan** |  |
| 1999-2005 | 29.17 (18.04 to 41.34) |
| 2005-2020 | 5.79 (4.67 to 6.92) |
| **Northeast** |  |
| 1999-2021 | 11.91 (10.97 to 12.86) |
| 2021-2023 | −3.42(−19.18 to 15.42) |
| **Midwest** |  |
| 1999-2002 | 42.63 (4.34 to 94.97) |
| 2002-2021 | 10.35 (9.48 to 11.23) |
| 2021-2023 | −2.19 (−15.7 to 13.55) |
| **South** |  |
| 1999-2006 | 21.12 (14.75 to 27.84) |
| 2006-2015 | 4.34 (1.62 to 7.12) |
| 2015-2021 | 17.06 (12.79 to 21.5) |
| 2021-2023 | 0.05 (−12.47 to 14.37) |
| **West** |  |
| 1999-2009 | 10.72 (7.2 to 14.35) |
| 2009-2018 | 1.54 (−1.58 to 4.76) |
| 2018-2023 | 20.04 (14.81 to 25.52) |
| **Age 15-44 Years** |  |
| 1999-2018 | 7.81 (6.67 to 8.96) |
| 2018-2021 | 21.2 (−2.05 to 49.98) |
| 2021-2023 | −2.7 (−18.49 to 16.12) |
| **Age 45-64 Years** |  |
| **1999-2007** | 16.12 (12.88 to 19.46) |
| 2007-2018 | 6.68 (5.55 to 7.82) |
| 2018-2021 | 18.25 (6.74 to 31) |
| 2021-2023 | 2.94 (−5.54 to 12.19) |
| **Age 65+ Years** |  |
| 1999-2019 | 11.34 (10.4 to 12.29) |
| 2019-2023 | 17.28 (12.99 to 21.73) |

APC = annual percent change; NH = non-Hispanic.

**Supplemental Table 3:** Overall and Sex‐Stratified Cardiovascular and Opioids Overdose–related Age-Adjusted Mortality Rates per 100,000 in Adults in the United States, 1999 to 2023.

| **Year** | **Overall** | **Women** | **Men** |
| --- | --- | --- | --- |
| 1999 | 0.36 (0.34-0.39) | 0.18 (0.15-0.2) | 0.57 (0.52-0.61) |
| 2000 | 0.39 (0.36-0.41) | 0.21 (0.18-0.23) | 0.58 (0.53-0.62) |
| 2001 | 0.42 (0.39-0.45) | 0.22 (0.19-0.24) | 0.64 (0.59-0.69) |
| 2002 | 0.64 (0.61-0.68) | 0.38 (0.35-0.42) | 0.94 (0.88-1) |
| 2003 | 0.74 (0.71-0.78) | 0.45 (0.41-0.48) | 1.07 (1.01-1.13) |
| 2004 | 0.83 (0.79-0.87) | 0.47 (0.43-0.51) | 1.17 (1.1-1.23) |
| 2005 | 0.86 (0.82-0.9) | 0.48 (0.44-0.52) | 1.27 (1.2-1.33) |
| 2006 | 1 (0.9-1.04) | 0.65 (0.6-0.69) | 1.44 (1.3-1.5) |
| 2007 | 1.1 (1.05-1.14) | 0.65 (0.61-0.7) | 1.54 (1.4-1.61) |
| 2008 | 1.18 (1.14-1.23) | 0.69 (0.65-0.74) | 1.71 (1.63-1.7) |
| 2009 | 1.4 (1.35-1.45) | 0.83 (0.78-0.88) | 1.93 (1.85-2.01) |
| 2010 | 1.38 (1.3-1.43) | 0.86 (0.81-0.91) | 1.92 (1.84-2) |
| 2011 | 1.47 (1.42-1.5) | 0.91 (0.85-0.96) | 2.04 (1.96-2.12) |
| 2012 | 1.47 (1.4-1.51) | 0.91 (0.85-0.96) | 2.04 (1.96-2.13) |
| 2013 | 1.57 (1.5-1.6) | 1 (0.94-1.05) | 2.11 (2.02-2.19) |
| 2014 | 1.8 (1.74-1.8) | 1.17 (1.1-1.2) | 2.46 (2.37-2.55) |
| 2015 | 1.8 (1.7-1.8) | 1.14 (1.08-1.19) | 2.5 (2.41-2.59) |
| 2016 | 2.12 (2.07-2.18) | 1.28 (1.22-1.34) | 3 (2.9-3.1) |
| 2017 | 2.4 (2.34-2.46) | 1.41 (1.35-1.47) | 3.38 (3.2-3.4) |
| 2018 | 2.42 (2.3-2.4) | 1.41 (1.34-1.48) | 3.5 (3.39-3.6) |
| 2019 | 2.56 (2.4-2.62) | 1.42 (1.35-1.48) | 3.74 (3.64-3.8) |
| 2020 | 3.38 (3.3-3.45) | 1.74 (1.67-1.81) | 4.99 (4.8-5.1) |
| 2021 | 4.1 (4.02-4.18) | 2.11 (2.03-2.19) | 6.05 (5.91-6.18) |
| 2022 | 4.1 (4.02-4.18) | 2.06 (1.98-2.14) | 6.13 (6-6.27) |
| 2023 | 4.08 (4.01-4.16) | 2.09 (2.01-2.17) | 6.13 (5.9-6.26) |

**Supplemental Table 4** Cardiovascular and Opioids Overdose –related Age-Adjusted Mortality Rates per 100,000, Stratified by Race in Adults in the United States, 1999 to 2023.

| **Year** | **NH White** | **NH Black** | **Hispanic/ Latino** |
| --- | --- | --- | --- |
| 1999 | 0.4 (0.37-0.43) | 0.33 (0.26-0.41) | 0.45 (0.35-0.54) |
| 2000 | 0.43 (0.4-0.46) | 0.35 (0.27-0.43) | 0.37 (0.29-0.46) |
| 2001 | 0.46 (0.42-0.49) | 0.39 (0.31-0.46) | 0.29 (0.23-0.38) |
| 2002 | 0.72 (0.68-0.76) | 0.55 (0.46-0.64) | 0.48 (0.38-0.57) |
| 2003 | 0.89 (0.85-0.94) | 0.52 (0.43-0.61) | 0.46 (0.37-0.55) |
| 2004 | 0.99 (0.94-1.04) | 0.53 (0.44-0.62) | 0.38 (0.31-0.48) |
| 2005 | 1.04 (0.99-1.08) | 0.56 (0.47-0.65) | 0.45 (0.37-0.53) |
| 2006 | 1.26 (1.21-1.32) | 0.68 (0.58-0.78) | 0.53 (0.44-0.62) |
| 2007 | 1.39 (1.34-1.45) | 0.67 (0.58-0.77) | 0.57 (0.48-0.66) |
| 2008 | 1.49 (1.43-1.55) | 0.73 (0.64-0.83) | 0.62 (0.53-0.72) |
| 2009 | 1.74 (1.68-1.81) | 0.76 (0.66-0.86) | 0.7 (0.61-0.79) |
| 2010 | 1.78 (1.72-1.85) | 0.79 (0.69-0.89) | 0.64 (0.55-0.73) |
| 2011 | 1.9 (1.83-1.97) | 0.76 (0.66-0.86) | 0.72 (0.63-0.81) |
| 2012 | 1.85 (1.78-1.92) | 0.88 (0.78-0.99) | 0.82 (0.72-0.91) |
| 2013 | 1.94 (1.87-2) | 1.06 (0.95-1.18) | 0.97 (0.86-1.07) |
| 2014 | 2.28 (2.21-2.36) | 1.39 (1.26-1.52) | 0.84 (0.75-0.93) |
| 2015 | 2.31 (2.24-2.39) | 1.34 (1.21-1.46) | 0.87 (0.77-0.96) |
| 2016 | 2.71 (2.63-2.8) | 1.83 (1.68-1.98) | 0.94 (0.84-1.03) |
| 2017 | 3.03 (2.95-3.12) | 2.35 (2.18-2.52) | 1.08 (0.98-1.18) |
| 2018 | 2.96 (2.88-3.05) | 2.67 (2.5-2.85) | 1.26 (1.15-1.37) |
| 2019 | 2.99 (2.9-3.07) | 3.37 (3.18-3.57) | 1.45 (1.34-1.56) |
| 2020 | 3.9 (3.8-4) | 4.43 (4.21-4.66) | 1.9 (1.7-2.03) |
| 2021 | 4.6 (4.49-4.71) | 6.03 (5.76-6.29) | 2.34 (2.2-2.49) |
| 2022 | 4.53 (4.42-4.64) | 6.16 (5.89-6.43) | 2.54 (2.39-2.69) |
| 2023 | 4.33 (4.22-4.43) | 6.48 (6.21-6.75) | 2.78 (2.63-2.93) |

NH = non-Hispanic.

**Supplemental Table 5** Cardiovascular and Opioids Overdose –related Age-Adjusted Mortality Rates per 100,000, Stratified by Census Region in Adults in the United States, 1999 to 2023.

| **Census Region** | **Year** | **Age Adjusted Mortality Rate (95% CI)** |
| --- | --- | --- |
| Northeast | 1999 | 0.28 (0.23-0.33) |
| Northeast | 2000 | 0.27 (0.22-0.32) |
| Northeast | 2001 | 0.29 (0.24-0.34) |
| Northeast | 2002 | 0.43 (0.37-0.49) |
| Northeast | 2003 | 0.49 (0.42-0.56) |
| Northeast | 2004 | 0.49 (0.42-0.56) |
| Northeast | 2005 | 0.62 (0.55-0.7) |
| Northeast | 2006 | 0.72 (0.64-0.8) |
| Northeast | 2007 | 0.72 (0.64-0.8) |
| Northeast | 2008 | 0.86 (0.77-0.94) |
| Northeast | 2009 | 0.85 (0.7-0.93) |
| Northeast | 2010 | 0.94 (0.85-1.03) |
| Northeast | 2011 | 1.05 (0.9-1.15) |
| Northeast | 2012 | 1.12 (1.02-1.22) |
| Northeast | 2013 | 1.19 (1.09-1.2) |
| Northeast | 2014 | 1.48 (1.3-1.59) |
| Northeast | 2015 | 1.59 (1.47-1.71) |
| Northeast | 2016 | 2.22 (2.08-2.36) |
| Northeast | 2017 | 2.6 (2.4-2.75) |
| Northeast | 2018 | 2.43 (2.2-2.5) |
| Northeast | 2019 | 2.46 (2.3-2.6) |
| Northeast | 2020 | 3 (2.8-3.1) |
| Northeast | 2021 | 3.32 (3.1-3.4) |
| Northeast | 2022 | 3.56 (3.3-3.7) |
| Northeast | 2023 | 3.07 (2.9-3.2) |
| Midwest | 1999 | 0.18 (0.1-0.2) |
| Midwest | 2000 | 0.3 (0.25-0.35) |
| Midwest | 2001 | 0.36 (0.31-0.41) |
| Midwest | 2002 | 0.54 (0.4-0.61) |
| Midwest | 2003 | 0.53 (0.47-0.59) |
| Midwest | 2004 | 0.67 (0.6-0.74) |
| Midwest | 2005 | 0.68 (0.61-0.75) |
| Midwest | 2006 | 0.86 (0.78-0.94) |
| Midwest | 2007 | 0.91 (0.83-0.99) |
| Midwest | 2008 | 0.97 (0.89-1.06) |
| Midwest | 2009 | 1.26 (1.1-1.3) |
| Midwest | 2010 | 1.29 (1.19-1.39) |
| Midwest | 2011 | 1.29 (1.19-1.39) |
| Midwest | 2012 | 1.34 (1.24-1.4) |
| Midwest | 2013 | 1.51 (1.41-1.62) |
| Midwest | 2014 | 1.87 (1.76-1.99) |
| Midwest | 2015 | 2.07 (1.94-2.19) |
| Midwest | 2016 | 2.39 (2.26-2.53) |
| Midwest | 2017 | 2.48 (2.34-2.61) |
| Midwest | 2018 | 2.37 (2.23-2.5) |
| Midwest | 2019 | 2.67 (2.53-2.81) |
| Midwest | 2020 | 3.25 (3.1-3.41) |
| Midwest | 2021 | 3.72 (3.55-3.88) |
| Midwest | 2022 | 3.51 (3.3-3.6) |
| Midwest | 2023 | 3.4 (3.2-3.5) |
| South | 1999 | 0.25 (0.2-0.29) |
| South | 2000 | 0.32 (0.28-0.35) |
| South | 2001 | 0.44 (0.39-0.48) |
| South | 2002 | 0.51 (0.46-0.56) |
| South | 2003 | 0.73 (0.67-0.79) |
| South | 2004 | 0.85 (0.79-0.91) |
| South | 2005 | 0.87 (0.8-0.93) |
| South | 2006 | 1.07 (1-1.14) |
| South | 2007 | 1.14 (1.07-1.21) |
| South | 2008 | 1.16 (1.09-1.23) |
| South | 2009 | 1.33 (1.25-1.41) |
| South | 2010 | 1.38 (1.3-1.46) |
| South | 2011 | 1.43 (1.35-1.51) |
| South | 2012 | 1.39 (1.31-1.46) |
| South | 2013 | 1.41 (1.33-1.48) |
| South | 2014 | 1.68 (1.6-1.77) |
| South | 2015 | 1.65 (1.57-1.74) |
| South | 2016 | 2.03 (1.94-2.12) |
| South | 2017 | 2.39 (2.29-2.49) |
| South | 2018 | 2.61 (2.51-2.71) |
| South | 2019 | 2.63 (2.53-2.73) |
| South | 2020 | 3.54 (3.42-3.66) |
| South | 2021 | 4.51 (4.37-4.64) |
| South | 2022 | 4.43 (4.29-4.56) |
| South | 2023 | 4.15 (4.02-4.28) |
| West | 1999 | 0.86 (0.78-0.94) |
| West | 2000 | 0.68 (0.61-0.78) |
| West | 2001 | 0.53 (0.47-0.6) |
| West | 2002 | 1.15 (1.05-1.24) |
| West | 2003 | 1.18 (1.08-1.27) |
| West | 2004 | 1.16 (1.07-1.25) |
| West | 2005 | 1.24 (1.14-1.33) |
| West | 2006 | 1.38 (1.28-1.47) |
| West | 2007 | 1.58 (1.48-1.69) |
| West | 2008 | 1.73 (1.63-1.84) |
| West | 2009 | 2.04 (1.93-2.16) |
| West | 2010 | 1.94 (1.82-2.05) |
| West | 2011 | 2.06 (1.94-2.18) |
| West | 2012 | 2.09 (1.98-2.21) |
| West | 2013 | 2.12 (2-2.23) |
| West | 2014 | 2.2 (2.08-2.32) |
| West | 2015 | 2.07 (1.95-2.18) |
| West | 2016 | 2.04 (1.92-2.15) |
| West | 2017 | 2.19 (2.07-2.31) |
| West | 2018 | 2.21 (2.09-2.33) |
| West | 2019 | 2.52 (2.39-2.64) |
| West | 2020 | 3.47 (3.32-3.62) |
| West | 2021 | 4.31 (4.14-4.48) |
| West | 2022 | 4.5 (4.33-4.67) |
| West | 2023 | 5.41 (5.22-5.59) |

**Supplemental Table 6** Cardiovascular and Opioids Overdose –related Mortality, Stratified by Place of Death in Adults in the United States, 1999 to 2023.

| **Place of Death** | **Deaths** |
| --- | --- |
| Medical Facility - Inpatient | 10,918 |
| Medical Facility - Outpatient or ER | 15,166 |
| Medical Facility - Dead on Arrival | 1,564 |
| Medical Facility - Status unknown | 11 |
| Decedent's home | 62,092 |
| Hospice facility | 267 |
| Nursing home/long term care | 414 |
| Other | 21,768 |
| Place of death unknown | 227 |
| **Total** | 112,427 |

**Supplemental Table 7** Cardiovascular and Opioids Overdose –related Age-Adjusted Mortality Rates per 100,000, Stratified by Urban-Rural Classification in Adults in the United States, 1999 to 2020.

| **Year** | **Metropolitan** | **Non-metropolitan** |
| --- | --- | --- |
| 1999 | 0.41 (0.38-0.44) | 0.24 (0.19-0.3) |
| 2000 | 0.42 (0.39-0.45) | 0.33 (0.27-0.39) |
| 2001 | 0.4 (0.37-0.43) | 0.49 (0.41-0.56) |
| 2002 | 0.67 (0.63-0.71) | 0.53 (0.45-0.61) |
| 2003 | 0.72 (0.69-0.76) | 0.79 (0.69-0.88) |
| 2004 | 0.78 (0.74-0.82) | 1.03 (0.92-1.14) |
| 2005 | 0.83 (0.79-0.87) | 1.1 (0.98-1.21) |
| 2006 | 0.99 (0.95-1.04) | 1.27 (1.15-1.39) |
| 2007 | 1.09 (1.04-1.13) | 1.25 (1.13-1.37) |
| 2008 | 1.17 (1.12-1.22) | 1.39 (1.27-1.52) |
| 2009 | 1.34 (1.29-1.39) | 1.68 (1.54-1.82) |
| 2010 | 1.34 (1.29-1.39) | 1.73 (1.59-1.87) |
| 2011 | 1.42 (1.37-1.47) | 1.96 (1.81-2.11) |
| 2012 | 1.4 (1.35-1.45) | 1.85 (1.7-1.99) |
| 2013 | 1.52 (1.46-1.57) | 1.83 (1.69-1.98) |
| 2014 | 1.72 (1.67-1.78) | 2.19 (2.03-2.35) |
| 2015 | 1.76 (1.71-1.82) | 2.16 (2.01-2.32) |
| 2016 | 2.09 (2.03-2.15) | 2.39 (2.22-2.56) |
| 2017 | 2.37 (2.31-2.44) | 2.54 (2.36-2.71) |
| 2018 | 2.45 (2.38-2.51) | 2.35 (2.19-2.52) |
| 2019 | 2.6 (2.54-2.67) | 2.27 (2.11-2.44) |
| 2020 | 3.4 (3.33-3.48) | 3.23 (3.03-3.42) |

**Supplemental Table 8** Cardiovascular and Opioids Overdose –related Crude Mortality Rates per 100,000, Stratified by Age Groups in Adults in the United States, 1999 to 2023.

| **Year** | **15-44 Years** | **45-64 Years** | **65+ Years** |
| --- | --- | --- | --- |
| 1999 | 0.36 (0.33-0.39) | 0.54 (0.48-0.59) | 0.15 (0.11-0.2) |
| 2000 | 0.33 (0.3-0.36) | 0.65 (0.58-0.71) | 0.14 (0.1-0.18) |
| 2001 | 0.36 (0.33-0.4) | 0.69 (0.63-0.76) | 0.11 (0.08-0.15) |
| 2002 | 0.56 (0.52-0.6) | 1.03 (0.95-1.11) | 0.21 (0.17-0.27) |
| 2003 | 0.61 (0.57-0.65) | 1.21 (1.13-1.3) | 0.25 (0.2-0.31) |
| 2004 | 0.68 (0.64-0.73) | 1.32 (1.24-1.41) | 0.25 (0.2-0.3) |
| 2005 | 0.7 (0.65-0.75) | 1.44 (1.36-1.53) | 0.29 (0.23-0.34) |
| 2006 | 0.86 (0.81-0.91) | 1.65 (1.56-1.74) | 0.3 (0.25-0.36) |
| 2007 | 0.87 (0.82-0.92) | 1.89 (1.8-1.99) | 0.35 (0.29-0.41) |
| 2008 | 0.93 (0.88-0.99) | 2 (1.9-2.1) | 0.42 (0.35-0.48) |
| 2009 | 1.06 (1-1.11) | 2.36 (2.26-2.47) | 0.5 (0.43-0.57) |
| 2010 | 1.11 (1.05-1.17) | 2.39 (2.29-2.5) | 0.37 (0.31-0.43) |
| 2011 | 1.12 (1.06-1.18) | 2.51 (2.41-2.62) | 0.56 (0.49-0.63) |
| 2012 | 1.07 (1.01-1.12) | 2.6 (2.49-2.71) | 0.59 (0.52-0.66) |
| 2013 | 1.09 (1.03-1.15) | 2.84 (2.72-2.95) | 0.62 (0.55-0.69) |
| 2014 | 1.34 (1.28-1.4) | 3.12 (3-3.24) | 0.74 (0.66-0.82) |
| 2015 | 1.33 (1.27-1.4) | 3.15 (3.03-3.27) | 0.86 (0.78-0.95) |
| 2016 | 1.63 (1.56-1.7) | 3.67 (3.54-3.8) | 0.83 (0.75-0.91) |
| 2017 | 1.9 (1.83-1.98) | 3.94 (3.81-4.07) | 1 (0.91-1.09) |
| 2018 | 1.86 (1.79-1.94) | 4.14 (4-4.27) | 1.14 (1.05-1.23) |
| 2019 | 2 (1.93-2.08) | 4.3 (4.16-4.44) | 1.27 (1.17-1.36) |
| 2020 | 2.75 (2.66-2.84) | 5.47 (5.31-5.63) | 1.41 (1.32-1.51) |
| 2021 | 3.32 (3.22-3.42) | 6.77 (6.59-6.94) | 1.8 (1.69-1.91) |
| 2022 | 3.2 (3.1-3.3) | 6.89 (6.71-7.07) | 2.01 (1.9-2.13) |
| 2023 | 3.1 (3.01-3.2) | 6.95 (6.77-7.13) | 2.35 (2.22-2.47) |
